# Ethyl 2-amino-6-(4-bromo­phen­yl)-4-(4-fluoro­phen­yl)cyclo­hexa-1,3-diene-1-carboxyl­ate

**DOI:** 10.1107/S1600536813023325

**Published:** 2013-08-23

**Authors:** B. Narayana, M. Sapnakumari, Jerry P. Jasinski, Peter M. Fraiser, H. S. Yathirajan

**Affiliations:** a Department of Studies in Chemistry, Mangalore University, Mangalagangotri 574 199, India; b Department of Chemistry, Keene State College, 229 Main Street, Keene, NH 03435-2001, USA; c Department of Studies in Chemistry, University of Mysore, Manasagangotri, Mysore 570 006, India

## Abstract

In the title compound, C_21_H_19_BrFNO_2_, two independent mol­ecules crystallize in the asymmetric unit. The cyclo­hexa-1,3-diene ring is in a slightly distorted screw-boat conformation. The dihedral angles between the mean planes of the 4-bromo­phenyl and 4-fluoro­phenyl rings are 81.0 (3) and 76.4 (2)° in the two independent mol­ecules. In the crystal, N—H⋯O hydrogen bonds link the molecules into [100] chains.

## Related literature

For cyclo­hexenones as precursors for functionalized derivatives, see: Samshuddin *et al.* (2013 ▶); For 4-bromo-4′-fluoro­chalcone derivatives, see: Fun *et al.* (2012*a* ▶,*b* ▶,*c* ▶). For related structures, see: Jasinski *et al.* (2012 ▶); Kant *et al.* (2012 ▶). For puckering parameters, see: Cremer & Pople (1975 ▶). For standard bond lengths, see Allen *et al.* (1987 ▶).
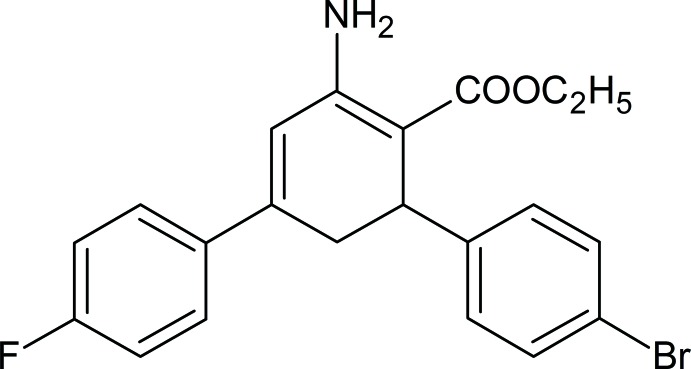


## Experimental

### 

#### Crystal data

C_21_H_19_BrFNO_2_
*M* *_r_* = 416.28
Monoclinic, 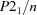

*a* = 9.4599 (2) Å
*b* = 23.3634 (5) Å
*c* = 17.2312 (4) Å
β = 96.001 (2)°
*V* = 3787.51 (15) Å^3^
*Z* = 8
Cu *K*α radiation
μ = 3.16 mm^−1^
*T* = 173 K
0.32 × 0.28 × 0.22 mm

#### Data collection

Agilent Xcalibur (Eos, Gemini) diffractometer
Absorption correction: multi-scan (*CrysAlis PRO* and *CrysAlis RED*; Agilent, 2012 ▶) *T*_min_ = 0.587, *T*_max_ = 1.000
25170 measured reflections
7427 independent reflections
5973 reflections with *I* > 2σ(*I*)
*R*_int_ = 0.040

#### Refinement

*R*[*F* ^2^ > 2σ(*F* ^2^)] = 0.051
*wR*(*F* ^2^) = 0.148
*S* = 1.03
7427 reflections
487 parameters
H atoms treated by a mixture of independent and constrained refinement
Δρ_max_ = 1.13 e Å^−3^
Δρ_min_ = −0.53 e Å^−3^

### 

Data collection: *CrysAlis PRO* (Agilent, 2012 ▶); cell refinement: *CrysAlis PRO*; data reduction: *CrysAlis RED* (Agilent, 2012 ▶); program(s) used to solve structure: *SUPERFLIP* (Palatinus & Chapuis, 2007 ▶); program(s) used to refine structure: *SHELXL2012* (Sheldrick, 2008 ▶); molecular graphics: *XP* in *SHELXTL* (Sheldrick, 2008 ▶); software used to prepare material for publication: *OLEX2* (Dolomanov *et al.*, 2009 ▶).

## Supplementary Material

Crystal structure: contains datablock(s) I. DOI: 10.1107/S1600536813023325/hg5341sup1.cif

Structure factors: contains datablock(s) I. DOI: 10.1107/S1600536813023325/hg5341Isup2.hkl

Supplementary material file. DOI: 10.1107/S1600536813023325/hg5341Isup3.cml

Additional supplementary materials:  crystallographic information; 3D view; checkCIF report

## Figures and Tables

**Table 1:** Hydrogen-bond geometry (Å, °)

*D*—H⋯*A*	*D*—H	H⋯*A*	*D*⋯*A*	*D*—H⋯*A*
N1*A*—H1*AA*⋯O2*A*	0.77 (3)	2.09 (3)	2.698 (4)	136 (3)
N1*A*—H1*AB*⋯O2*B* ^i^	0.77 (4)	2.17 (4)	2.915 (4)	162 (4)
N1*B*—H1*BA*⋯O2*A* ^ii^	0.87 (3)	2.20 (4)	3.044 (3)	163 (3)
N1*B*—H1*BB*⋯O2*B*	0.85 (4)	2.07 (4)	2.714 (3)	132 (3)

Symmetry codes: (i) 
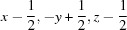
; (ii) 
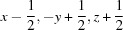
.

**Fig. 1.**
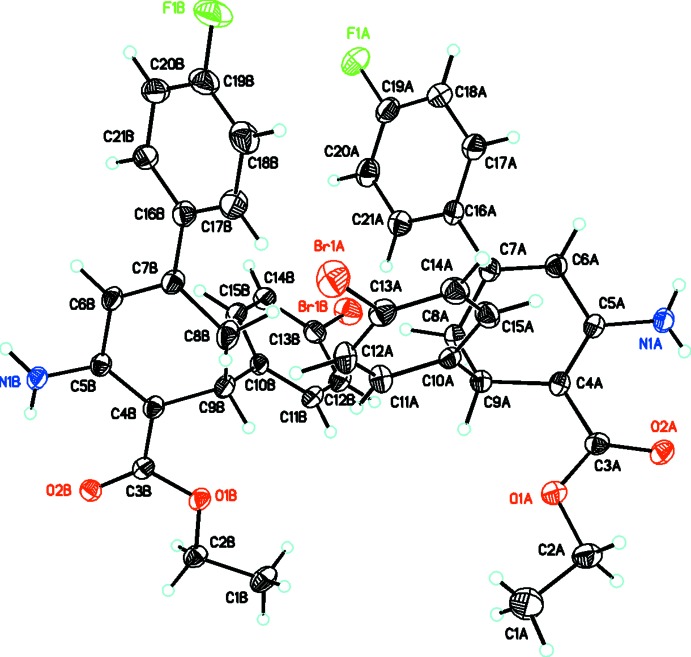
Molecular structure of the title compound showing the atom labeling scheme and 30% probability displacement ellipsoids.

**Fig. 2.**
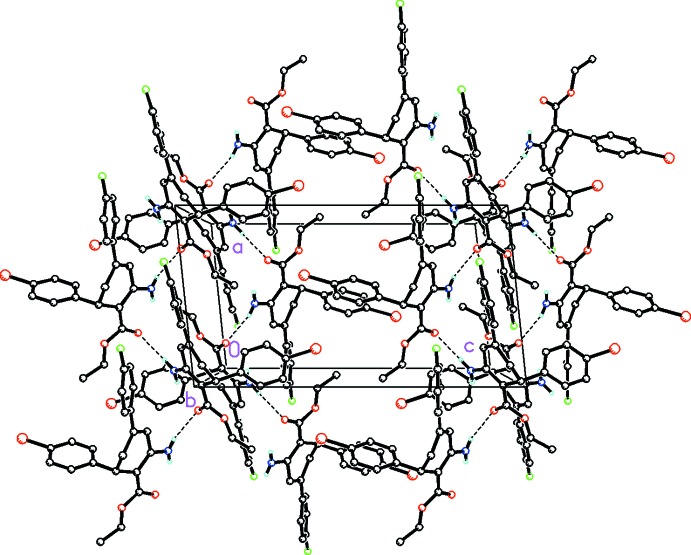
Packing diagram of the title compound viewed along the b axis. Dashed lines indicate weak N—H···O hydrogen bonds which form chains along [100]. H atoms not involved in these hydrogen bonds have been deleted for clarity.

**Fig. 3.**
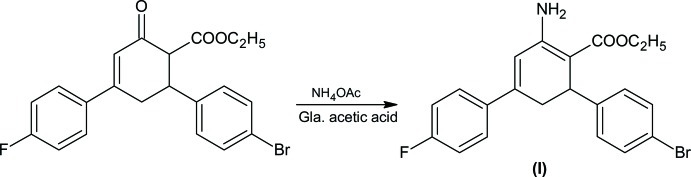
Synthesis of (I).

## supplementary crystallographic information

### 1. Comment

Cyclohexenones are well known precursors for various functionalized derivatives such as indazoles, dibenzodiazepines, terphenyls, aminated derivatves etc. (Samshuddin *et al.*, 2013). The title compound, (I), was prepared by the amination of cyclohexenone derivative of 4-bromo-4'-fluorochalcone with ammonium acetate. The crystal structure of a similar aminated product viz., ethyl 2-amino-4,6-bis (4-fluorophenyl)cyclohexa-1,3-diene-1-carboxylate has been reported (Jasinski *et al.*, 2012). Also, the crystal structure of precursor of the title compound (I) viz., ethyl 6-(4-bromophenyl)-4- (4-fluorophenyl)-2-oxocyclohex-3-ene-1-carboxylate has been reported (Kant *et al.*, 2012). In continuation of our work on the synthesis of 4-bromo-4'-fluorochalcone derivatives (Fun *et al.* 2012*a*, 2012*b*, 2012*c*), the title compound (I), C_21_H_19_NO_2_FBr, was prepared and its crystal structure is reported.

In the title compound, C_21_H_19_NO_2_FBr two independent molecules [A & B]crystallize in the asymmetric unit (Fig. 1). The cyclohexa-1,3-diene ring is in a slightly distorted screw-boat conformation (puckering parameters Q, θ, and φ = 0.434 (3)Å, 64.7 (4)°, 271.3 (4)° [A] and 0.355 (3)Å, 63.3 (5)°, 269.7 (6)° [B], respectively; (Cremer & Pople, 1975). Bond lengths are in normal ranges (Allen *et al.*, 1987)). The dihedral angle between the mean planes of the 4-bromophenyl and 4-fluorophenyl rings is 81.0 (3)° [A] and 76.4 (2)° [B]. In the crystal, N—H···O hydrogen bonds form chains along [010] and contribute to packing stability (Fig. 2).

### 2. Experimental

A mixture of ethyl 6-(4-bromophenyl)-4-(4-fluorophenyl)-2-oxocyclohex- 3-ene-1-carboxylate (4.17 g, 0.01 mol) and ammonium acetate (1.54 g, 0.02 mol) in 30 ml glacial acetic acid was refluxed for 6 hours. The reaction mixture was cooled and poured into 50 ml ice-cold water. The precipitate was collected by filtration and purified by recrystallization from ethanol. Single crystals were grown from ethanol by the slow evaporation method. (m.p. 418–420 K).

### 3. Refinement

H1AA, H1AB, H1BA and H1BB were located by a difference map and refined isotropically. All of the remaining H atoms were placed in their calculated positions and then refined using the riding model with Atom—H lengths of 0.95Å (CH) or 0.99° (CH_2_) or 0.98° (CH_3_). Isotropic displacement parameters for these atoms were set to 1.2 (CH) or 1.5 (OH) times *U*_eq_ of the parent atom. Idealised Me refined as rotating group: C1A(H1AC,H1AD,H1AE), C1B(H1BC,H1BD,H1BE).

### Crystal data

**Table:**

C_21_H_19_BrFNO_2_	*F*(000) = 1696
*M**_r_* = 416.28	*D*_x_ = 1.460 Mg m^−^^3^
Monoclinic, *P*2_1_/*n*	Cu *K*α radiation, λ = 1.5418 Å
*a* = 9.4599 (2) Å	Cell parameters from 7321 reflections
*b* = 23.3634 (5) Å	θ = 3.2–72.3°
*c* = 17.2312 (4) Å	µ = 3.16 mm^−^^1^
β = 96.001 (2)°	*T* = 173 K
*V* = 3787.51 (15) Å^3^	Irregular, yellow
*Z* = 8	0.32 × 0.28 × 0.22 mm

### Data collection

**Table:**

Agilent Xcalibur (Eos, Gemini) diffractometer	7427 independent reflections
Radiation source: Enhance (Cu) X-ray Source	5973 reflections with *I* > 2σ(*I*)
Graphite monochromator	*R*_int_ = 0.040
Detector resolution: 16.0416 pixels mm^-1^	θ_max_ = 72.5°, θ_min_ = 3.2°
ω scans	*h* = −11→10
Absorption correction: multi-scan (*CrysAlis PRO* and *CrysAlis RED*; Agilent, 2012)	*k* = −28→25
*T*_min_ = 0.587, *T*_max_ = 1.000	*l* = −21→20
25170 measured reflections	

### Refinement

**Table:**

Refinement on *F*^2^	Primary atom site location: structure-invariant direct methods
Least-squares matrix: full	Hydrogen site location: mixed
*R*[*F*^2^ > 2σ(*F*^2^)] = 0.051	H atoms treated by a mixture of independent and constrained refinement
*wR*(*F*^2^) = 0.148	*w* = 1/[σ^2^(*F*_o_^2^) + (0.0771*P*)^2^ + 2.9658*P*] where *P* = (*F*_o_^2^ + 2*F*_c_^2^)/3
*S* = 1.03	(Δ/σ)_max_ = 0.001
7427 reflections	Δρ_max_ = 1.13 e Å^−^^3^
487 parameters	Δρ_min_ = −0.53 e Å^−^^3^
0 restraints	

### Special details

**Table:**

Geometry. All esds (except the esd in the dihedral angle between two l.s. planes) are estimated using the full covariance matrix. The cell esds are taken into account individually in the estimation of esds in distances, angles and torsion angles; correlations between esds in cell parameters are only used when they are defined by crystal symmetry. An approximate (isotropic) treatment of cell esds is used for estimating esds involving l.s. planes.

### Fractional atomic coordinates and isotropic or equivalent isotropic displacement parameters (Å^2^)

**Table:**

	*x*	*y*	*z*	*U*_iso_*/*U*_eq_	
Br1A	0.37157 (5)	0.43234 (2)	0.64815 (2)	0.05829 (15)	
F1A	−0.1959 (2)	0.20568 (10)	0.24677 (16)	0.0692 (7)	
O1A	0.7906 (2)	0.41473 (10)	0.33655 (13)	0.0414 (5)	
O2A	0.7261 (2)	0.46881 (9)	0.23026 (12)	0.0364 (5)	
N1A	0.4536 (3)	0.45651 (12)	0.16870 (16)	0.0377 (6)	
H1AA	0.523 (4)	0.4738 (13)	0.1701 (18)	0.022 (8)*	
H1AB	0.382 (4)	0.4657 (16)	0.147 (2)	0.039 (10)*	
C1A	1.0010 (5)	0.4226 (2)	0.4216 (3)	0.0819 (16)	
H1AC	1.0218	0.3825	0.4110	0.123*	
H1AD	0.9448	0.4249	0.4662	0.123*	
H1AE	1.0903	0.4436	0.4337	0.123*	
C2A	0.9191 (4)	0.44839 (17)	0.3518 (2)	0.0478 (8)	
H2AA	0.8955	0.4888	0.3620	0.057*	
H2AB	0.9752	0.4471	0.3064	0.057*	
C3A	0.6981 (3)	0.43038 (12)	0.27564 (17)	0.0317 (6)	
C4A	0.5687 (3)	0.39797 (12)	0.27159 (16)	0.0315 (6)	
C5A	0.4532 (3)	0.41309 (12)	0.22021 (16)	0.0315 (6)	
C6A	0.3182 (3)	0.38225 (13)	0.22013 (17)	0.0342 (6)	
H6A	0.2340	0.3988	0.1947	0.041*	
C7A	0.3106 (3)	0.33092 (12)	0.25538 (16)	0.0325 (6)	
C8A	0.4461 (3)	0.30516 (12)	0.29415 (17)	0.0345 (6)	
H8AA	0.4924	0.2827	0.2552	0.041*	
H8AB	0.4227	0.2786	0.3357	0.041*	
C9A	0.5506 (3)	0.35068 (12)	0.32998 (16)	0.0320 (6)	
H9A	0.6451	0.3316	0.3416	0.038*	
C10A	0.5058 (3)	0.37256 (12)	0.40751 (16)	0.0303 (6)	
C11A	0.5381 (4)	0.33980 (13)	0.47408 (18)	0.0385 (7)	
H11A	0.5885	0.3049	0.4704	0.046*	
C12A	0.4988 (4)	0.35662 (14)	0.54587 (18)	0.0436 (7)	
H12A	0.5218	0.3337	0.5910	0.052*	
C13A	0.4255 (3)	0.40728 (14)	0.55037 (18)	0.0388 (7)	
C14A	0.3931 (4)	0.44123 (15)	0.4856 (2)	0.0418 (7)	
H14A	0.3431	0.4762	0.4897	0.050*	
C15A	0.4342 (3)	0.42383 (14)	0.41475 (18)	0.0391 (7)	
H15A	0.4130	0.4474	0.3701	0.047*	
C16A	0.1765 (3)	0.29819 (13)	0.25295 (17)	0.0348 (6)	
C17A	0.0446 (4)	0.32440 (14)	0.2399 (2)	0.0452 (8)	
H17A	0.0400	0.3646	0.2319	0.054*	
C18A	−0.0808 (4)	0.29345 (16)	0.2383 (2)	0.0500 (8)	
H18A	−0.1703	0.3122	0.2308	0.060*	
C19A	−0.0729 (4)	0.23585 (16)	0.2475 (2)	0.0472 (8)	
C20A	0.0542 (4)	0.20668 (15)	0.2570 (2)	0.0518 (9)	
H20A	0.0565	0.1661	0.2612	0.062*	
C21A	0.1786 (4)	0.23819 (14)	0.2602 (2)	0.0443 (7)	
H21A	0.2674	0.2189	0.2674	0.053*	
Br1B	0.32238 (5)	0.06632 (2)	0.13555 (2)	0.06171 (15)	
F1B	−0.1976 (3)	0.29436 (12)	0.58030 (17)	0.0815 (8)	
O1B	0.7450 (2)	0.08834 (9)	0.46357 (12)	0.0365 (5)	
O2B	0.7067 (2)	0.02371 (9)	0.55597 (12)	0.0341 (4)	
N1B	0.4602 (3)	0.03596 (11)	0.62187 (15)	0.0357 (5)	
H1BA	0.394 (4)	0.0269 (14)	0.6509 (19)	0.030 (8)*	
H1BB	0.532 (4)	0.0150 (15)	0.618 (2)	0.036 (9)*	
C1B	0.9329 (4)	0.09062 (15)	0.3842 (2)	0.0476 (8)	
H1BC	0.8671	0.0900	0.3364	0.071*	
H1BD	1.0235	0.0733	0.3740	0.071*	
H1BE	0.9490	0.1303	0.4014	0.071*	
C2B	0.8696 (3)	0.05690 (13)	0.4476 (2)	0.0387 (7)	
H2BA	0.8437	0.0177	0.4292	0.046*	
H2BB	0.9381	0.0542	0.4950	0.046*	
C3B	0.6680 (3)	0.06649 (12)	0.51815 (17)	0.0311 (6)	
C4B	0.5413 (3)	0.09959 (12)	0.52506 (17)	0.0322 (6)	
C5B	0.4440 (3)	0.08185 (12)	0.57450 (17)	0.0315 (6)	
C6B	0.3117 (3)	0.11419 (12)	0.57836 (17)	0.0353 (6)	
H6B	0.2381	0.0980	0.6048	0.042*	
C7B	0.2919 (3)	0.16682 (12)	0.54492 (18)	0.0367 (6)	
C8B	0.4108 (4)	0.19339 (14)	0.5065 (2)	0.0478 (8)	
H8BA	0.4680	0.2174	0.5454	0.057*	
H8BB	0.3694	0.2190	0.4643	0.057*	
C9B	0.5104 (3)	0.15061 (12)	0.47171 (18)	0.0344 (6)	
H9B	0.6029	0.1709	0.4692	0.041*	
C10B	0.4599 (3)	0.13196 (12)	0.38868 (18)	0.0338 (6)	
C11B	0.5379 (3)	0.14645 (15)	0.32743 (19)	0.0439 (8)	
H11B	0.6200	0.1698	0.3375	0.053*	
C12B	0.4985 (4)	0.12771 (17)	0.2523 (2)	0.0492 (8)	
H12B	0.5529	0.1379	0.2110	0.059*	
C13B	0.3789 (4)	0.09399 (14)	0.23795 (19)	0.0425 (7)	
C14B	0.2974 (4)	0.07961 (14)	0.2971 (2)	0.0427 (7)	
H14B	0.2139	0.0571	0.2864	0.051*	
C15B	0.3393 (3)	0.09842 (13)	0.3717 (2)	0.0408 (7)	
H15B	0.2843	0.0882	0.4126	0.049*	
C16B	0.1623 (3)	0.20068 (13)	0.55263 (18)	0.0383 (7)	
C17B	0.1648 (4)	0.26074 (15)	0.5510 (2)	0.0506 (9)	
H17B	0.2508	0.2799	0.5432	0.061*	
C18B	0.0451 (5)	0.29259 (17)	0.5605 (2)	0.0583 (10)	
H18B	0.0479	0.3332	0.5602	0.070*	
C19B	−0.0789 (4)	0.26373 (18)	0.5706 (2)	0.0545 (9)	
C20B	−0.0863 (4)	0.20618 (17)	0.5734 (2)	0.0496 (8)	
H20B	−0.1734	0.1877	0.5806	0.060*	
C21B	0.0355 (3)	0.17440 (14)	0.56566 (19)	0.0412 (7)	
H21B	0.0319	0.1339	0.5693	0.049*	

### Atomic displacement parameters (Å^2^)

**Table:**

	*U*^11^	*U*^22^	*U*^33^	*U*^12^	*U*^13^	*U*^23^
Br1A	0.0728 (3)	0.0676 (3)	0.0371 (2)	0.0088 (2)	0.01827 (18)	−0.00322 (16)
F1A	0.0491 (12)	0.0690 (15)	0.0906 (18)	−0.0199 (11)	0.0129 (12)	0.0164 (13)
O1A	0.0323 (11)	0.0490 (12)	0.0426 (12)	−0.0040 (9)	0.0018 (9)	0.0067 (10)
O2A	0.0354 (11)	0.0427 (11)	0.0327 (10)	−0.0033 (9)	0.0117 (8)	0.0009 (9)
N1A	0.0364 (15)	0.0392 (14)	0.0367 (14)	−0.0076 (13)	−0.0002 (12)	0.0096 (11)
C1A	0.062 (3)	0.101 (4)	0.076 (3)	−0.025 (3)	−0.023 (2)	0.025 (3)
C2A	0.0348 (17)	0.057 (2)	0.050 (2)	−0.0069 (15)	−0.0012 (14)	0.0017 (16)
C3A	0.0294 (14)	0.0369 (15)	0.0303 (14)	0.0033 (11)	0.0090 (11)	−0.0031 (11)
C4A	0.0355 (15)	0.0318 (14)	0.0280 (13)	−0.0030 (12)	0.0068 (11)	0.0012 (11)
C5A	0.0356 (15)	0.0307 (14)	0.0290 (14)	−0.0035 (11)	0.0076 (11)	0.0007 (11)
C6A	0.0345 (15)	0.0361 (15)	0.0316 (14)	−0.0031 (12)	0.0016 (11)	0.0028 (11)
C7A	0.0360 (15)	0.0329 (14)	0.0287 (14)	−0.0028 (12)	0.0044 (11)	0.0001 (11)
C8A	0.0404 (16)	0.0305 (14)	0.0320 (14)	−0.0013 (12)	0.0015 (12)	0.0031 (11)
C9A	0.0315 (14)	0.0330 (14)	0.0313 (14)	−0.0011 (11)	0.0027 (11)	0.0031 (11)
C10A	0.0251 (13)	0.0332 (14)	0.0325 (14)	−0.0048 (11)	0.0025 (11)	0.0024 (11)
C11A	0.0459 (17)	0.0357 (15)	0.0339 (15)	0.0017 (13)	0.0038 (13)	0.0040 (12)
C12A	0.056 (2)	0.0441 (17)	0.0304 (15)	0.0007 (15)	0.0056 (14)	0.0080 (13)
C13A	0.0393 (16)	0.0478 (17)	0.0303 (15)	−0.0025 (14)	0.0080 (12)	−0.0032 (13)
C14A	0.0397 (17)	0.0468 (18)	0.0393 (17)	0.0067 (14)	0.0061 (13)	−0.0005 (13)
C15A	0.0404 (17)	0.0451 (17)	0.0315 (15)	0.0060 (13)	0.0018 (12)	0.0059 (12)
C16A	0.0393 (16)	0.0341 (15)	0.0314 (14)	−0.0049 (12)	0.0050 (12)	0.0039 (11)
C17A	0.0445 (18)	0.0381 (16)	0.054 (2)	−0.0030 (14)	0.0088 (15)	0.0052 (14)
C18A	0.0397 (18)	0.056 (2)	0.056 (2)	0.0000 (15)	0.0092 (15)	0.0112 (17)
C19A	0.0426 (18)	0.053 (2)	0.0471 (19)	−0.0129 (15)	0.0082 (15)	0.0070 (15)
C20A	0.058 (2)	0.0392 (18)	0.059 (2)	−0.0102 (16)	0.0086 (17)	0.0055 (15)
C21A	0.0453 (18)	0.0407 (17)	0.0469 (18)	−0.0027 (14)	0.0053 (14)	0.0029 (14)
Br1B	0.0622 (3)	0.0741 (3)	0.0478 (2)	0.0055 (2)	0.00066 (19)	−0.00554 (18)
F1B	0.0622 (15)	0.0926 (19)	0.0917 (19)	0.0427 (14)	0.0173 (14)	0.0110 (15)
O1B	0.0350 (11)	0.0394 (11)	0.0377 (11)	0.0048 (9)	0.0154 (9)	0.0039 (9)
O2B	0.0336 (10)	0.0355 (10)	0.0332 (10)	0.0024 (8)	0.0039 (8)	0.0014 (8)
N1B	0.0362 (13)	0.0371 (13)	0.0358 (13)	0.0046 (11)	0.0140 (11)	0.0068 (10)
C1B	0.0456 (18)	0.0488 (19)	0.052 (2)	−0.0019 (15)	0.0248 (16)	−0.0068 (16)
C2B	0.0348 (15)	0.0390 (16)	0.0440 (17)	0.0022 (13)	0.0127 (13)	−0.0027 (13)
C3B	0.0321 (14)	0.0334 (14)	0.0283 (14)	−0.0037 (11)	0.0057 (11)	−0.0051 (11)
C4B	0.0351 (15)	0.0297 (14)	0.0332 (14)	0.0009 (12)	0.0106 (11)	−0.0002 (11)
C5B	0.0356 (15)	0.0300 (13)	0.0299 (14)	−0.0001 (11)	0.0081 (11)	−0.0007 (11)
C6B	0.0390 (16)	0.0334 (14)	0.0359 (15)	0.0025 (12)	0.0150 (12)	0.0038 (12)
C7B	0.0390 (16)	0.0303 (14)	0.0430 (16)	0.0029 (12)	0.0144 (13)	0.0004 (12)
C8B	0.058 (2)	0.0322 (16)	0.058 (2)	0.0094 (15)	0.0280 (17)	0.0110 (14)
C9B	0.0356 (15)	0.0287 (14)	0.0415 (16)	0.0016 (12)	0.0160 (12)	0.0051 (12)
C10B	0.0326 (14)	0.0303 (14)	0.0403 (16)	0.0085 (11)	0.0120 (12)	0.0120 (12)
C11B	0.0329 (15)	0.057 (2)	0.0435 (18)	−0.0055 (14)	0.0102 (13)	0.0132 (15)
C12B	0.0404 (18)	0.068 (2)	0.0408 (18)	−0.0018 (16)	0.0120 (14)	0.0133 (16)
C13B	0.0422 (17)	0.0464 (17)	0.0391 (17)	0.0081 (14)	0.0045 (13)	0.0049 (14)
C14B	0.0372 (16)	0.0399 (16)	0.0515 (19)	−0.0001 (13)	0.0075 (14)	0.0062 (14)
C15B	0.0378 (16)	0.0385 (16)	0.0482 (18)	0.0005 (13)	0.0148 (14)	0.0095 (13)
C16B	0.0437 (17)	0.0360 (15)	0.0367 (16)	0.0048 (13)	0.0112 (13)	0.0015 (12)
C17B	0.055 (2)	0.0398 (17)	0.059 (2)	0.0078 (16)	0.0197 (17)	0.0024 (15)
C18B	0.071 (3)	0.046 (2)	0.060 (2)	0.0206 (19)	0.015 (2)	0.0042 (17)
C19B	0.051 (2)	0.067 (2)	0.047 (2)	0.0254 (19)	0.0099 (16)	0.0043 (17)
C20B	0.0385 (17)	0.070 (2)	0.0410 (18)	0.0084 (16)	0.0059 (14)	0.0041 (16)
C21B	0.0407 (17)	0.0443 (17)	0.0391 (16)	0.0046 (14)	0.0073 (13)	0.0021 (13)

### Geometric parameters (Å, º)

**Table:**

Br1A—C13A	1.903 (3)	Br1B—C13B	1.903 (3)
F1A—C19A	1.359 (4)	F1B—C19B	1.357 (4)
O1A—C2A	1.449 (4)	O1B—C2B	1.439 (4)
O1A—C3A	1.345 (4)	O1B—C3B	1.348 (3)
O2A—C3A	1.237 (4)	O2B—C3B	1.228 (4)
N1A—H1AA	0.77 (3)	N1B—H1BA	0.87 (3)
N1A—H1AB	0.77 (4)	N1B—H1BB	0.85 (4)
N1A—C5A	1.348 (4)	N1B—C5B	1.346 (4)
C1A—H1AC	0.9800	C1B—H1BC	0.9800
C1A—H1AD	0.9800	C1B—H1BD	0.9800
C1A—H1AE	0.9800	C1B—H1BE	0.9800
C1A—C2A	1.489 (6)	C1B—C2B	1.519 (4)
C2A—H2AA	0.9900	C2B—H2BA	0.9900
C2A—H2AB	0.9900	C2B—H2BB	0.9900
C3A—C4A	1.434 (4)	C3B—C4B	1.442 (4)
C4A—C5A	1.379 (4)	C4B—C5B	1.382 (4)
C4A—C9A	1.516 (4)	C4B—C9B	1.515 (4)
C5A—C6A	1.466 (4)	C5B—C6B	1.469 (4)
C6A—H6A	0.9500	C6B—H6B	0.9500
C6A—C7A	1.350 (4)	C6B—C7B	1.363 (4)
C7A—C8A	1.507 (4)	C7B—C8B	1.498 (4)
C7A—C16A	1.478 (4)	C7B—C16B	1.477 (4)
C8A—H8AA	0.9900	C8B—H8BA	0.9900
C8A—H8AB	0.9900	C8B—H8BB	0.9900
C8A—C9A	1.537 (4)	C8B—C9B	1.537 (4)
C9A—H9A	1.0000	C9B—H9B	1.0000
C9A—C10A	1.531 (4)	C9B—C10B	1.524 (4)
C10A—C11A	1.386 (4)	C10B—C11B	1.391 (4)
C10A—C15A	1.388 (4)	C10B—C15B	1.389 (4)
C11A—H11A	0.9500	C11B—H11B	0.9500
C11A—C12A	1.385 (4)	C11B—C12B	1.381 (5)
C12A—H12A	0.9500	C12B—H12B	0.9500
C12A—C13A	1.378 (5)	C12B—C13B	1.379 (5)
C13A—C14A	1.377 (5)	C13B—C14B	1.382 (5)
C14A—H14A	0.9500	C14B—H14B	0.9500
C14A—C15A	1.381 (5)	C14B—C15B	1.377 (5)
C15A—H15A	0.9500	C15B—H15B	0.9500
C16A—C17A	1.388 (5)	C16B—C17B	1.404 (5)
C16A—C21A	1.407 (4)	C16B—C21B	1.387 (5)
C17A—H17A	0.9500	C17B—H17B	0.9500
C17A—C18A	1.387 (5)	C17B—C18B	1.379 (5)
C18A—H18A	0.9500	C18B—H18B	0.9500
C18A—C19A	1.356 (5)	C18B—C19B	1.379 (6)
C19A—C20A	1.378 (5)	C19B—C20B	1.348 (6)
C20A—H20A	0.9500	C20B—H20B	0.9500
C20A—C21A	1.384 (5)	C20B—C21B	1.389 (5)
C21A—H21A	0.9500	C21B—H21B	0.9500
			
C3A—O1A—C2A	117.3 (2)	C3B—O1B—C2B	116.9 (2)
H1AA—N1A—H1AB	125 (4)	H1BA—N1B—H1BB	122 (3)
C5A—N1A—H1AA	116 (2)	C5B—N1B—H1BA	120 (2)
C5A—N1A—H1AB	118 (3)	C5B—N1B—H1BB	117 (2)
H1AC—C1A—H1AD	109.5	H1BC—C1B—H1BD	109.5
H1AC—C1A—H1AE	109.5	H1BC—C1B—H1BE	109.5
H1AD—C1A—H1AE	109.5	H1BD—C1B—H1BE	109.5
C2A—C1A—H1AC	109.5	C2B—C1B—H1BC	109.5
C2A—C1A—H1AD	109.5	C2B—C1B—H1BD	109.5
C2A—C1A—H1AE	109.5	C2B—C1B—H1BE	109.5
O1A—C2A—C1A	106.4 (3)	O1B—C2B—C1B	105.4 (3)
O1A—C2A—H2AA	110.4	O1B—C2B—H2BA	110.7
O1A—C2A—H2AB	110.4	O1B—C2B—H2BB	110.7
C1A—C2A—H2AA	110.4	C1B—C2B—H2BA	110.7
C1A—C2A—H2AB	110.4	C1B—C2B—H2BB	110.7
H2AA—C2A—H2AB	108.6	H2BA—C2B—H2BB	108.8
O1A—C3A—C4A	112.1 (2)	O1B—C3B—C4B	111.6 (2)
O2A—C3A—O1A	121.7 (3)	O2B—C3B—O1B	121.7 (3)
O2A—C3A—C4A	126.2 (3)	O2B—C3B—C4B	126.7 (3)
C3A—C4A—C9A	120.5 (3)	C3B—C4B—C9B	118.8 (2)
C5A—C4A—C3A	120.7 (3)	C5B—C4B—C3B	120.2 (3)
C5A—C4A—C9A	118.4 (3)	C5B—C4B—C9B	120.7 (3)
N1A—C5A—C4A	124.0 (3)	N1B—C5B—C4B	124.9 (3)
N1A—C5A—C6A	115.6 (3)	N1B—C5B—C6B	115.2 (3)
C4A—C5A—C6A	120.4 (3)	C4B—C5B—C6B	119.9 (3)
C5A—C6A—H6A	119.2	C5B—C6B—H6B	119.2
C7A—C6A—C5A	121.5 (3)	C7B—C6B—C5B	121.7 (3)
C7A—C6A—H6A	119.2	C7B—C6B—H6B	119.2
C6A—C7A—C8A	118.2 (3)	C6B—C7B—C8B	119.0 (3)
C6A—C7A—C16A	122.2 (3)	C6B—C7B—C16B	121.6 (3)
C16A—C7A—C8A	119.6 (3)	C16B—C7B—C8B	119.1 (3)
C7A—C8A—H8AA	109.1	C7B—C8B—H8BA	108.5
C7A—C8A—H8AB	109.1	C7B—C8B—H8BB	108.5
C7A—C8A—C9A	112.5 (2)	C7B—C8B—C9B	115.0 (3)
H8AA—C8A—H8AB	107.8	H8BA—C8B—H8BB	107.5
C9A—C8A—H8AA	109.1	C9B—C8B—H8BA	108.5
C9A—C8A—H8AB	109.1	C9B—C8B—H8BB	108.5
C4A—C9A—C8A	110.5 (2)	C4B—C9B—C8B	111.3 (2)
C4A—C9A—H9A	107.3	C4B—C9B—H9B	106.5
C4A—C9A—C10A	113.4 (2)	C4B—C9B—C10B	111.5 (2)
C8A—C9A—H9A	107.3	C8B—C9B—H9B	106.5
C10A—C9A—C8A	110.9 (2)	C10B—C9B—C8B	114.0 (3)
C10A—C9A—H9A	107.3	C10B—C9B—H9B	106.5
C11A—C10A—C9A	118.7 (3)	C11B—C10B—C9B	120.2 (3)
C11A—C10A—C15A	118.0 (3)	C15B—C10B—C9B	121.9 (3)
C15A—C10A—C9A	123.3 (3)	C15B—C10B—C11B	117.8 (3)
C10A—C11A—H11A	119.1	C10B—C11B—H11B	119.3
C12A—C11A—C10A	121.7 (3)	C12B—C11B—C10B	121.5 (3)
C12A—C11A—H11A	119.1	C12B—C11B—H11B	119.3
C11A—C12A—H12A	120.7	C11B—C12B—H12B	120.5
C13A—C12A—C11A	118.5 (3)	C13B—C12B—C11B	119.0 (3)
C13A—C12A—H12A	120.7	C13B—C12B—H12B	120.5
C12A—C13A—Br1A	119.8 (2)	C12B—C13B—Br1B	120.4 (3)
C14A—C13A—Br1A	118.8 (2)	C12B—C13B—C14B	121.2 (3)
C14A—C13A—C12A	121.3 (3)	C14B—C13B—Br1B	118.5 (3)
C13A—C14A—H14A	120.4	C13B—C14B—H14B	120.6
C13A—C14A—C15A	119.2 (3)	C15B—C14B—C13B	118.8 (3)
C15A—C14A—H14A	120.4	C15B—C14B—H14B	120.6
C10A—C15A—H15A	119.4	C10B—C15B—H15B	119.1
C14A—C15A—C10A	121.2 (3)	C14B—C15B—C10B	121.8 (3)
C14A—C15A—H15A	119.4	C14B—C15B—H15B	119.1
C17A—C16A—C7A	122.1 (3)	C17B—C16B—C7B	121.2 (3)
C17A—C16A—C21A	117.3 (3)	C21B—C16B—C7B	121.2 (3)
C21A—C16A—C7A	120.6 (3)	C21B—C16B—C17B	117.5 (3)
C16A—C17A—H17A	119.1	C16B—C17B—H17B	119.3
C18A—C17A—C16A	121.8 (3)	C18B—C17B—C16B	121.4 (4)
C18A—C17A—H17A	119.1	C18B—C17B—H17B	119.3
C17A—C18A—H18A	120.7	C17B—C18B—H18B	121.0
C19A—C18A—C17A	118.6 (3)	C17B—C18B—C19B	118.1 (4)
C19A—C18A—H18A	120.7	C19B—C18B—H18B	121.0
F1A—C19A—C20A	118.7 (3)	F1B—C19B—C18B	118.9 (4)
C18A—C19A—F1A	118.5 (3)	C20B—C19B—F1B	118.3 (4)
C18A—C19A—C20A	122.8 (3)	C20B—C19B—C18B	122.7 (3)
C19A—C20A—H20A	121.0	C19B—C20B—H20B	120.6
C19A—C20A—C21A	118.0 (3)	C19B—C20B—C21B	118.9 (3)
C21A—C20A—H20A	121.0	C21B—C20B—H20B	120.6
C16A—C21A—H21A	119.3	C16B—C21B—C20B	121.3 (3)
C20A—C21A—C16A	121.5 (3)	C16B—C21B—H21B	119.3
C20A—C21A—H21A	119.3	C20B—C21B—H21B	119.3
			
Br1A—C13A—C14A—C15A	178.8 (3)	Br1B—C13B—C14B—C15B	178.6 (2)
F1A—C19A—C20A—C21A	−177.9 (3)	F1B—C19B—C20B—C21B	−178.3 (3)
O1A—C3A—C4A—C5A	170.7 (3)	O1B—C3B—C4B—C5B	175.9 (3)
O1A—C3A—C4A—C9A	−2.3 (4)	O1B—C3B—C4B—C9B	1.7 (4)
O2A—C3A—C4A—C5A	−8.9 (5)	O2B—C3B—C4B—C5B	−4.6 (5)
O2A—C3A—C4A—C9A	178.1 (3)	O2B—C3B—C4B—C9B	−178.8 (3)
N1A—C5A—C6A—C7A	165.1 (3)	N1B—C5B—C6B—C7B	168.0 (3)
C2A—O1A—C3A—O2A	6.0 (4)	C2B—O1B—C3B—O2B	4.4 (4)
C2A—O1A—C3A—C4A	−173.7 (3)	C2B—O1B—C3B—C4B	−176.1 (2)
C3A—O1A—C2A—C1A	179.5 (3)	C3B—O1B—C2B—C1B	179.4 (3)
C3A—C4A—C5A—N1A	3.0 (5)	C3B—C4B—C5B—N1B	3.5 (5)
C3A—C4A—C5A—C6A	−175.7 (3)	C3B—C4B—C5B—C6B	−176.9 (3)
C3A—C4A—C9A—C8A	−152.6 (3)	C3B—C4B—C9B—C8B	−157.6 (3)
C3A—C4A—C9A—C10A	82.2 (3)	C3B—C4B—C9B—C10B	73.9 (3)
C4A—C5A—C6A—C7A	−16.0 (4)	C4B—C5B—C6B—C7B	−11.6 (5)
C4A—C9A—C10A—C11A	−155.2 (3)	C4B—C9B—C10B—C11B	−118.5 (3)
C4A—C9A—C10A—C15A	24.8 (4)	C4B—C9B—C10B—C15B	58.9 (4)
C5A—C4A—C9A—C8A	34.3 (4)	C5B—C4B—C9B—C8B	28.3 (4)
C5A—C4A—C9A—C10A	−90.9 (3)	C5B—C4B—C9B—C10B	−100.2 (3)
C5A—C6A—C7A—C8A	−1.0 (4)	C5B—C6B—C7B—C8B	−2.8 (5)
C5A—C6A—C7A—C16A	−177.4 (3)	C5B—C6B—C7B—C16B	−176.5 (3)
C6A—C7A—C8A—C9A	33.8 (4)	C6B—C7B—C8B—C9B	29.7 (5)
C6A—C7A—C16A—C17A	−23.2 (5)	C6B—C7B—C16B—C17B	151.0 (3)
C6A—C7A—C16A—C21A	153.7 (3)	C6B—C7B—C16B—C21B	−25.7 (5)
C7A—C8A—C9A—C4A	−48.8 (3)	C7B—C8B—C9B—C4B	−40.7 (4)
C7A—C8A—C9A—C10A	77.8 (3)	C7B—C8B—C9B—C10B	86.4 (4)
C7A—C16A—C17A—C18A	−179.5 (3)	C7B—C16B—C17B—C18B	−178.0 (3)
C7A—C16A—C21A—C20A	−179.1 (3)	C7B—C16B—C21B—C20B	179.6 (3)
C8A—C7A—C16A—C17A	160.5 (3)	C8B—C7B—C16B—C17B	−22.7 (5)
C8A—C7A—C16A—C21A	−22.6 (4)	C8B—C7B—C16B—C21B	160.6 (3)
C8A—C9A—C10A—C11A	79.9 (3)	C8B—C9B—C10B—C11B	114.5 (3)
C8A—C9A—C10A—C15A	−100.2 (3)	C8B—C9B—C10B—C15B	−68.1 (4)
C9A—C4A—C5A—N1A	176.1 (3)	C9B—C4B—C5B—N1B	177.6 (3)
C9A—C4A—C5A—C6A	−2.7 (4)	C9B—C4B—C5B—C6B	−2.9 (4)
C9A—C10A—C11A—C12A	−179.0 (3)	C9B—C10B—C11B—C12B	176.7 (3)
C9A—C10A—C15A—C14A	178.5 (3)	C9B—C10B—C15B—C14B	−177.2 (3)
C10A—C11A—C12A—C13A	0.1 (5)	C10B—C11B—C12B—C13B	0.1 (5)
C11A—C10A—C15A—C14A	−1.5 (5)	C11B—C10B—C15B—C14B	0.3 (5)
C11A—C12A—C13A—Br1A	−179.3 (3)	C11B—C12B—C13B—Br1B	−179.1 (3)
C11A—C12A—C13A—C14A	−0.9 (5)	C11B—C12B—C13B—C14B	1.0 (5)
C12A—C13A—C14A—C15A	0.5 (5)	C12B—C13B—C14B—C15B	−1.5 (5)
C13A—C14A—C15A—C10A	0.8 (5)	C13B—C14B—C15B—C10B	0.8 (5)
C15A—C10A—C11A—C12A	1.0 (5)	C15B—C10B—C11B—C12B	−0.8 (5)
C16A—C7A—C8A—C9A	−149.8 (3)	C16B—C7B—C8B—C9B	−156.5 (3)
C16A—C17A—C18A—C19A	−1.9 (6)	C16B—C17B—C18B—C19B	−0.9 (6)
C17A—C16A—C21A—C20A	−2.0 (5)	C17B—C16B—C21B—C20B	2.8 (5)
C17A—C18A—C19A—F1A	179.2 (3)	C17B—C18B—C19B—F1B	179.8 (4)
C17A—C18A—C19A—C20A	−1.2 (6)	C17B—C18B—C19B—C20B	1.7 (6)
C18A—C19A—C20A—C21A	2.6 (6)	C18B—C19B—C20B—C21B	−0.2 (6)
C19A—C20A—C21A—C16A	−0.9 (5)	C19B—C20B—C21B—C16B	−2.1 (5)
C21A—C16A—C17A—C18A	3.5 (5)	C21B—C16B—C17B—C18B	−1.2 (6)

### Hydrogen-bond geometry (Å, º)

**Table:**

*D*—H···*A*	*D*—H	H···*A*	*D*···*A*	*D*—H···*A*
N1*A*—H1*AA*···O2*A*	0.77 (3)	2.09 (3)	2.698 (4)	136 (3)
N1*A*—H1*AB*···O2*B*^i^	0.77 (4)	2.17 (4)	2.915 (4)	162 (4)
N1*B*—H1*BA*···O2*A*^ii^	0.87 (3)	2.20 (4)	3.044 (3)	163 (3)
N1*B*—H1*BB*···O2*B*	0.85 (4)	2.07 (4)	2.714 (3)	132 (3)

Symmetry codes: (i) *x*−1/2, −*y*+1/2, *z*−1/2; (ii) *x*−1/2, −*y*+1/2, *z*+1/2.

## References

[bb1] Agilent (2012). *CrysAlis PRO* and *CrysAlis RED* Agilent Technologies, Yarnton, England.

[bb2] Allen, F. H., Kennard, O., Watson, D. G., Brammer, L., Orpen, A. G. & Taylor, R. (1987). *J. Chem. Soc. Perkin Trans. 2*, pp. S1–19.

[bb3] Cremer, D. & Pople, J. A. (1975). *J. Am. Chem. Soc.* **97**, 1354–1358.

[bb4] Dolomanov, O. V., Bourhis, L. J., Gildea, R. J., Howard, J. A. K. & Puschmann, H. (2009). *J. Appl. Cryst.* **42**, 339–341.

[bb5] Fun, H.-K., Chia, T. S., Sapnakumari, M., Narayana, B. & Sarojini, B. K. (2012*a*). *Acta Cryst.* E**68**, o2680. 10.1107/S160053681203454X PMC3435702 22969573

[bb6] Fun, H.-K., Loh, W.-S., Sapnakumari, M., Narayana, B. & Sarojini, B. K. (2012*b*). *Acta Cryst.* E**68**, o2655–o2656. 10.1107/S1600536812034368 PMC3435682 22969553

[bb7] Fun, H.-K., Loh, W.-S., Sapnakumari, M., Narayana, B. & Sarojini, B. K. (2012*c*). *Acta Cryst.* E**68**, o2586. 10.1107/S1600536812033351 PMC3415024 22905011

[bb8] Jasinski, J. P., Golen, J. A., Samshuddin, S., Narayana, B. & Yathirajan, H. S. (2012). *Acta Cryst.* E**68**, o585. 10.1107/S160053681200373X PMC3297311 22412501

[bb9] Kant, R., Gupta, V. K., Kapoor, K., Sapnakumari, M., Narayana, B. & Sarojini, B. K. (2012). *Acta Cryst.* E**68**, o2917–o2918. 10.1107/S1600536812038202 PMC3470264 23125708

[bb10] Palatinus, L. & Chapuis, G. (2007). *J. Appl. Cryst.* **40**, 786–790.

[bb11] Samshuddin, S., Narayana, B., Sarojini, B. K. & Madhu, L. N. (2013). *Med. Chem. Res.* **22**, 3002–3011.

[bb12] Sheldrick, G. M. (2008). *Acta Cryst.* A**64**, 112–122. 10.1107/S0108767307043930 18156677

